# Chemotherapy‐Associated Brain Morphological Alterations in Breast Cancer: Implications for Cognitive Decline and Neurodegenerative Risks

**DOI:** 10.1002/alz70856_103823

**Published:** 2025-12-24

**Authors:** Roberto Vicidomini, Rifa Sanjida Punnota, Paul Edison, Laura Kenny

**Affiliations:** ^1^ Imperial College London, London, Greater London, United Kingdom; ^2^ Cardiff University, Wales, United Kingdom

## Abstract

**Background:**

Chemotherapeutic agents are widely recognized for side effects intrinsic to their mechanisms of action. While advancements in cancer treatment have improved survival rates, they pose challenges regarding chemotherapy's long‐term impact on quality of life. Chemotherapy‐associated cognitive impairment (Chemobrain), affecting ∼33% of post‐chemotherapy breast cancer patients, stems from a multitude of concurrent factors^1^, leading to varying outcomes: while some patients experience a transient decline that resolves within 12 months, others face long‐lasting effects that may increase the risk of neurodegenerative conditions, including Alzheimer's Disease (AD). Investigating its causes and phenotype may enable early detection, proactive management, and pave the way for preventive strategies.

**Method:**

270 breast cancer patients, who received anthracycline‐taxane chemotherapy within the last 12 months, were enrolled following neurocognitive pre‐screening tests conducted on an Artificial Intelligence‐driven online platform. Among these, 18 patients with poorer pre‐screening scores, along with 19 controls, underwent in‐person neurocognitive assessments and an MRI scan (3‐Tesla). The acquired MRI images were analysed using Region of Interest (ROI) and Voxel‐Based Morphometric (VBM) group‐level analysis.

**Result:**

ROI analysis revealed that Chemobrain patients exhibited significantly lower grey matter volumes (mm^3^) in the left isthmus cingulate and pars opercularis regions, as well as in grey matter surface areas (mm^2^) in the same regions, along with the left medial orbitofrontal, right isthmus cingulate, lingual, and temporal pole areas. VBM analysis identified a statistically significant decrease in the periventricular areas, cingulate gyrus, precuneus, as well as in the parietal and medial frontal lobes, within patients treated with chemotherapy. Additionally, in‐person neurocognitive tests revealed significantly poorer semantic and verbal fluency and lower MMSE scores in Chemobrain patients.

**Conclusion:**

The deep brain regions showing significant differences between the two groups may align with those affected in other conditions, such as Small Vessels Disease and Vascular Dementia, with some belonging to the Default Mode Network, which is also compromised in AD. Chemotherapy‐induced neuroinflammation could potentially disrupt the neurovascular unit, induce vascular toxicity, and lead to brain morphological changes contributing to the cognitive impairment underlying the Chemobrain phenotype.

**Reference**

1. Fleming et al. “Cognitive impairment after cancer treatment: mechanisms, clinical characterization, and management” BMJ.2023Mar15;380:e071726

